# Newly identified breast luminal progenitor and gestational stem cell populations likely give rise to HER2-overexpressing and basal-like breast cancers

**DOI:** 10.1007/s12672-022-00500-6

**Published:** 2022-05-28

**Authors:** James R. W. McMullen, Ubaldo Soto

**Affiliations:** Department of Basic Sciences, School of Medicine, Loma Linda University, Loma Linda, CA 92350 USA

**Keywords:** Mammary gland development, Breast cancer, Basal-like breast cancer, HER2 breast cancer, Breast neoplasms

## Abstract

Breast Cancer (BrC) is a common malignancy with genetically diverse subtypes. There is evidence that specific BrC subtypes originate from particular normal mammary cell populations. However, the cell populations that give rise to most BrC subtypes are unidentified. Several human breast scRNAseq datasets are available. In this research, we utilized a robust human scRNAseq dataset to identify population-specific marker genes and then identified the expression of these marker genes in specific BrC subtypes. In humans, several BrC subtypes, HER2-enriched, basal-like, and triple-negative (TN), are more common in women who have had children. This observation suggests that cell populations that originate during pregnancy give rise to these BrCs. The current human datasets have few normal parous samples, so we supplemented this research with mouse datasets, which contain mammary cells from various developmental stages. This research identified two novel normal breast cell populations that may be the origin of the basal-like and HER2-overexpressing subtypes, respectively. A stem cell-like population, SC, that expresses gestation-specific genes has similar gene expression patterns to basal-like BrCs. A novel luminal progenitor cell population and HER2-overexpressing BrCs are marked by S100A7, S100A8, and S100A9 expression. We bolstered our findings by examining SC gene expression in TN BrC scRNAseq datasets and S100A7-A9 gene expression in BrC cell lines. We discovered that several potential cancer stem cell populations highly express most of the SC genes in TN BrCs and confirmed S100A8 and A9 overexpression in a HER2-overexpressing BrC cell line. In summary, normal SC and the novel luminal progenitor cell population likely give rise to basal-like and HER2-overexpressing BrCs, respectively. Characterizing these normal cell populations may facilitate a better understanding of specific BrCs subtypes.

**Supplementary information:**

The online version contains supplementary material available at 10.1007/s12672-022-00500-6.

## Introduction

Breast cancer (BrC) is the most common cancer and the second most common cause of cancer-related death in women [1, 2]. BrCs are categorized as Luminal A, Luminal B, HER2-enriched, and triple-negative (TN) for treatment purposes [3]. TN BrCs are heterogeneous, and researchers have used gene expression patterns to subdivide them into seven categories, including basal-like BrC [3, 4].

There is evidence that specific cell populations give rise to distinct BrC subtypes, but the origin of most BrC subtypes is unknown. Two research papers identified that luminal progenitor cells give rise or are associated with basal-like TN BrC [5, 6]. In mice, BRCA1 mutant mammary luminal progenitor cells can generate a tumor closely resembling the basal-like BrC subtype [5]. In human BRCA1 mutant basal-like cancers, there is an aberrant increase in the number of luminal progenitor cells [6]. The normal cell populations that become the other BrC subtypes are unknown.

Multiple scRNAseq datasets have provided plenty of information on mammary cell populations in humans and mice [7–11]. The human datasets are limited since they primarily focus on characterizing normal adult nulliparous cell populations [7, 8]. The mouse datasets examine the mammary tissue in multiple crucial developmental stages, including fetal and adult nulliparous, gestational, lactating, and post-involution [9, 10, 12]. The mouse datasets illustrate a considerable change in mammary cell populations with pregnancy. One surprising finding in the mouse is that nulliparous and post-involution mammary cell populations are different [9]. This feature is important because these different cell populations may become specific BrC subtypes in humans.

Nulliparous and parous women have different risks for BrC subtypes. Parous women have an increased risk of HER2-enriched, basal-like BrCs, and TN BrCs but reduced Luminal A and Luminal B tumor risk compared to nulliparous women [13–18]. The change in breast cancer risk led researchers to hypothesize that pregnancy changes human breast cell populations in normal development [19]. We now know this phenomenon occurs in mice, but it remains unproven in humans [9]. From the above data, we conclude that Luminal A and Luminal B cancers likely originate from nulliparous cell populations, while HER2-enriched and TN BrCs likely originate from post-pregnancy cell populations.

Since parous human breast cells are not well characterized while mouse mammary cells are, we wanted to use both the human and mouse datasets, but we needed to confirm that the mouse mammary gland is a good model of the human breast [9, 10]. Both mice and human mammary cells share the same eight general cell populations: stem cells, common progenitors, luminal progenitors, differentiated ductal cells, alveolar progenitors, differentiated alveolar cells, myoepithelial progenitors, and myoepithelial cells [20–22]. Further, the mouse and human mammary cell populations share multiple markers that identify these populations, including ITGA6, ESR1, PGR, KIT, CD29, and EpCAM, and even share differentiation patterns [8, 20–24].

We characterized normal human adult mammary cell populations from published data and identified marker genes for these populations. We then identified marker gene expression in human BrC subtypes and in the mouse mammary datasets. Lastly, based on shared gene expression patterns, we identified two potential cell populations of origin for human BrC subtypes.

## Materials and methods

### scRNA-seq bioinformatics

Patient samples from the GSE161529 dataset from Pal and colleagues were downloaded from the Gene Expression Omnibus (GEO) server [7]. We generated a folder containing a barcode, features, and matrix file for each patient sample. We imported seven normal patient sample datasets with the Read10X Seurat command in R. Patient samples N-N280-Epi, N-N1105-Epi, N-MH0064-Epi are premenopausal and nulliparous, N-MH0023-Epi is premenopausal and parous, N-PM0342-Epi is postmenopausal and nulliparous, and N-PM0372-Epi and N-MH275-Epi are postmenopausal and parous [7]. We removed potentially low-quality cells with ≥ 20% mitochondrial genes, < 500 genes, < 1500 unique molecular identifiers (UMI), or < 0.8 log10GenesPerUMI. We removed genes expressed in less than ten cells. SCTransform was used to normalize, scale, and find variable features in the data. Anchor-based integration was performed. We used 100 principal components for PCA analysis, followed by t-SNE and UMAP analysis. SCTransform is highly effective, so 100 principle components contribute to robust analysis. We performed a clustering analysis with several cluster resolution values and generated heatmaps, t-SNE, and dot plots. The top differentially expressed genes were used as population-specific marker genes and examined in detail. We also examined eight scRNAseq TN BrC datasets from GSE161529 using the above protocols, with the additional step of removing cell populations that highly express CD31 (endothelial cell marker) and CD45 (immune cell marker). Four samples had BRCA mutations: TN-B1-MH4031, TN-B1-MH0131, TN-B1-Tum0554, TN-B1-MH0177; “B1” indicates BRCA mutant. Four samples had normal BRCA: TN-MH0126, TN-MH0135, TN-SH0106, TN-MH0114-T2.

### Using GENT2 to identify marker gene expression in human breast cancers

We then examined the expression of the identified population-specific marker genes in human BrCs. The GENT2 database facilitated this analysis [25]. GENT2 collates and runs statistics on breast cancer microarray datasets from multiple groups in the GEO database.

### Identifying human population-specific marker gene expression patterns in mouse mammary datasets

Chung and colleagues and Bach and colleagues created user-friendly online resources to explore the RNA expression in mouse mammary gland cell populations in fetal and adult nulliparous, gestational, lactating, and post-involution glands [9, 10]. https://wahl-lab-salk.shinyapps.io/Mammary_snATAC and https://marionilab.cruk.cam.ac.uk/mammaryGland are the respective web addresses [9, 10]. We identified the mouse mammary cell populations that expressed the human population-specific marker genes.

### Cell lines

BT-474, MCF-7, MDA-MB-231, SKBR3, and Hs 578T BrC cell lines were from the American Type Culture Collection (Manassas, VA, USA, Cat# ATCC-HTB-20, ATCC-HTB-22, ATCC-HTB-26, ATCC-HTB-30, ATCC-HTB-126 respectively). Some of them were a kind gifts from Dr. Daisy De Leon lab (SKBR3) and Dr. Eileen Brantley lab (MCF-7, MDA-MB-231) at Loma Linda University. These BrC cell lines were cultured in RPMI (Genesee Scientific, San Diego, CA, USA) supplemented with 10% FBS (Genesee Scientific, San Diego, CA, USA) and penicillin/streptomycin (Sigma-Aldrich, St. Louis, MO, USA) on tissue culture treated plates at 37 ˚C under 5% CO_2_.

### Quantitative reverse transcription PCR

Quantitative reverse transcription PCR (RT-qPCR) was performed as described previously [26]. Briefly, total RNA was purified from cultured cells using the Quick-RNA Miniprep Kit (Zymo Research, Irvine, CA, USA) according to manufacturer’s instructions. RNA was quantified by measuring absorbance at 260 nm using the BioMate 3 spectrophotometer (Thermo Scientific, Waltham, MA, USA) and RNA purity was assessed using the 260 nm/280nm ratio. 1ug RNA was used for reverse transcription (RT) using Maxima H Minus Reverse Transcriptase (ThermoFisher Scientific, Waltham, MA, USA), 1 uL of 0.2 ug/uL primer “random” (Roche, Sigma-Aldrich, St. Louis, MO, USA), and 1uL of 10 mM dNTP (ThermoFisher Scientific, Waltham, MA, USA) in a 20 uL reaction according to manufacturer’s protocols. Quantitative PCR was performed with complementary DNA (cDNA) from the RT reaction and iTaq™ Universal SYBR^®^ Green Supermix (Bio-Rad, Hercules, CA, USA).

Gene-specific primers were designed using Primer-BLAST software (NIH). Primers for genes are listed in Supplementary Table S1. Quantitative real-time PCR analysis was performed using a CFX-96 PCR instrument (Bio-Rad, Hercules, CA, USA). Primers for genes indicated above were obtained from Integrated DNA Technologies (Coralville, IA, USA). Data was processed using CFX Maestro software (Bio-Rad, Hercules, CA, USA). 28 S rRNA, ACTB, B2M, and GAPDH expression levels were used to normalize the other gene expressions.

## Results

### Human mammary gland cells have population-specific gene markers

**Fig. 1:**
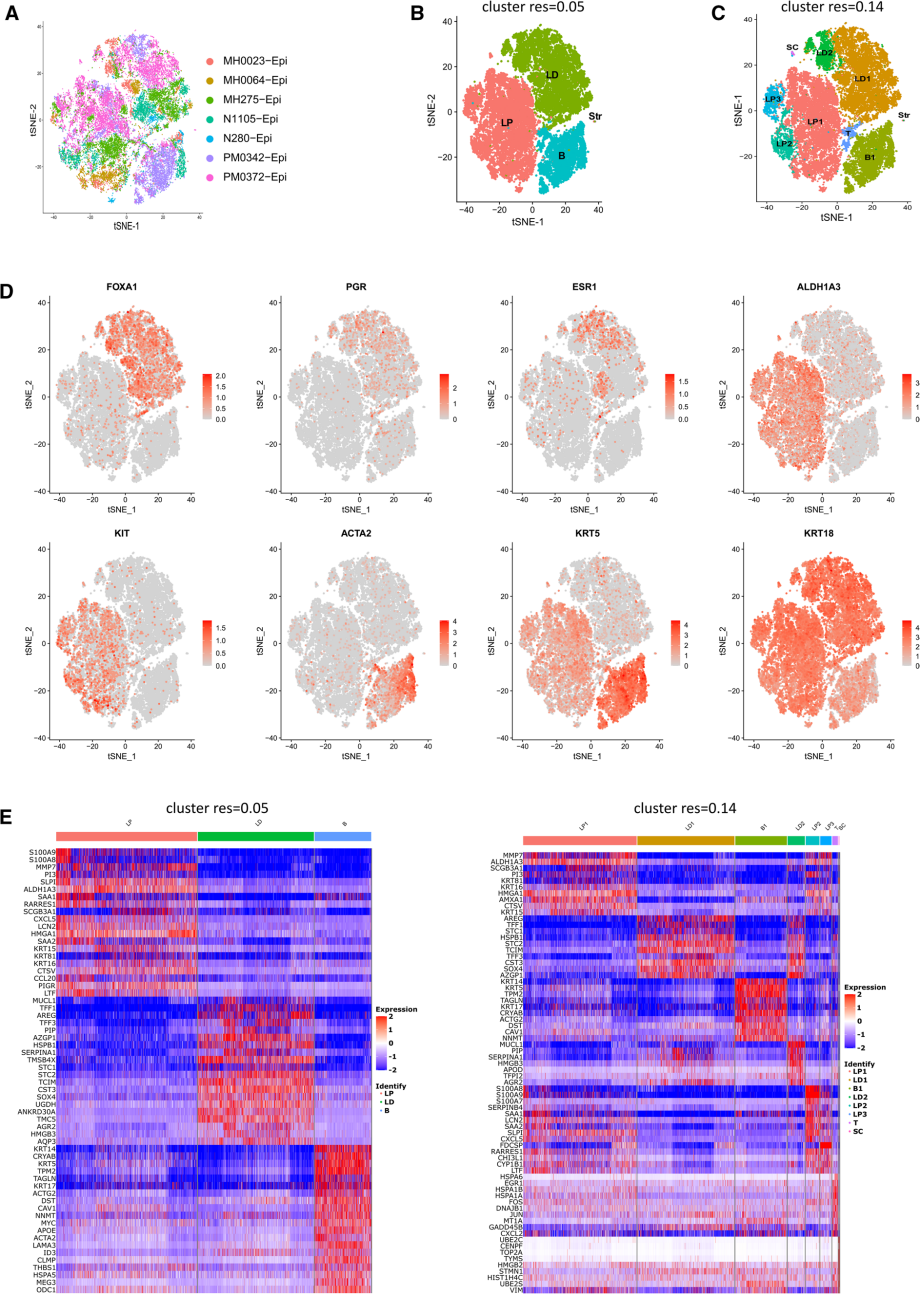
Human adult mammary cell populations.** A** t-SNE plot of seven normal breast scRNA-seq datasets. Individual dots correspond to cells and dot color indicates tissue sample. **B** Four cell clusters, luminal progenitor (LP), luminal differentiated (LD), basal (B), and stromal (Str) are identified at 0.05 cluster resolution in a t-SNE plot. **C** Eight cell clusters, three luminal progenitors (LP1-3), two luminal differentiated (LD1-2), a basal (B1), a stem cell like population (SC), a transition (T), and a stromal (Str) cell cluster are identified at 0.14 cluster resolution in a t-SNE plot. **D** Expression of LP, LD, and B marker genes was analyzed in t-SNE plots. Grey indicates low or no gene expression while red indicates high expression. **E** Heatmaps of the top 20 and 10 differentially expressed genes at the 0.05 and 0.14 cluster resolution, respectively. Red indicates high expression while blue indicates low expression

Pal and colleagues robustly analyzed human breast tissue in pre and postmenopausal states [7]. We reexamined seven normal patient samples from their dataset, GSE161529, looking for population-specific markers at several clustering resolutions. The cells from different patient samples are shown in a t-SNE plot with dots representing individual cells and dot color representing individual tissue samples (Fig. 1A). We tested multiple clustering resolutions and decided to focus on 0.05 and 0.14 clustering resolutions based on strong marker gene expression patterns (Fig. 1B, C). At 0.05 clustering resolution, we used known mammary epithelial cell population markers to identify and group the human cell populations into luminal progenitor (LP), luminal differentiated (LD), basal (B), and contaminate stromal (Str) cells (Fig. 1B). When we reexamined the cells at 0.14 cluster resolution, subpopulations were identified, including three luminal progenitors (LP1-3), two luminal differentiated (LD1-2), a basal (B1), a transition (T), a stem cell-like (SC), and a stromal cell (Str) population (Fig. 1C). The expression of the marker genes used to identify LP, LD, and B populations are shown in Fig. 1D. FOXA1, PGR (progesterone receptor), ESR1 (estrogen receptor) identify luminal differentiated cells [22]. ALDH1A3 and KIT marks luminal progenitor cells [23, 27]. ACTA2 (smooth muscle actin) and KRT5 mark basal cells [28]. Lastly, KRT18 is a general luminal marker identifying LP and LD [28]. We removed stromal cells from further analysis and made heatmaps for the top 20 and 10 differentially expressed genes at 0.05 and 0.14 cluster resolutions, respectively (Fig. 1E).

**Fig. 2:**
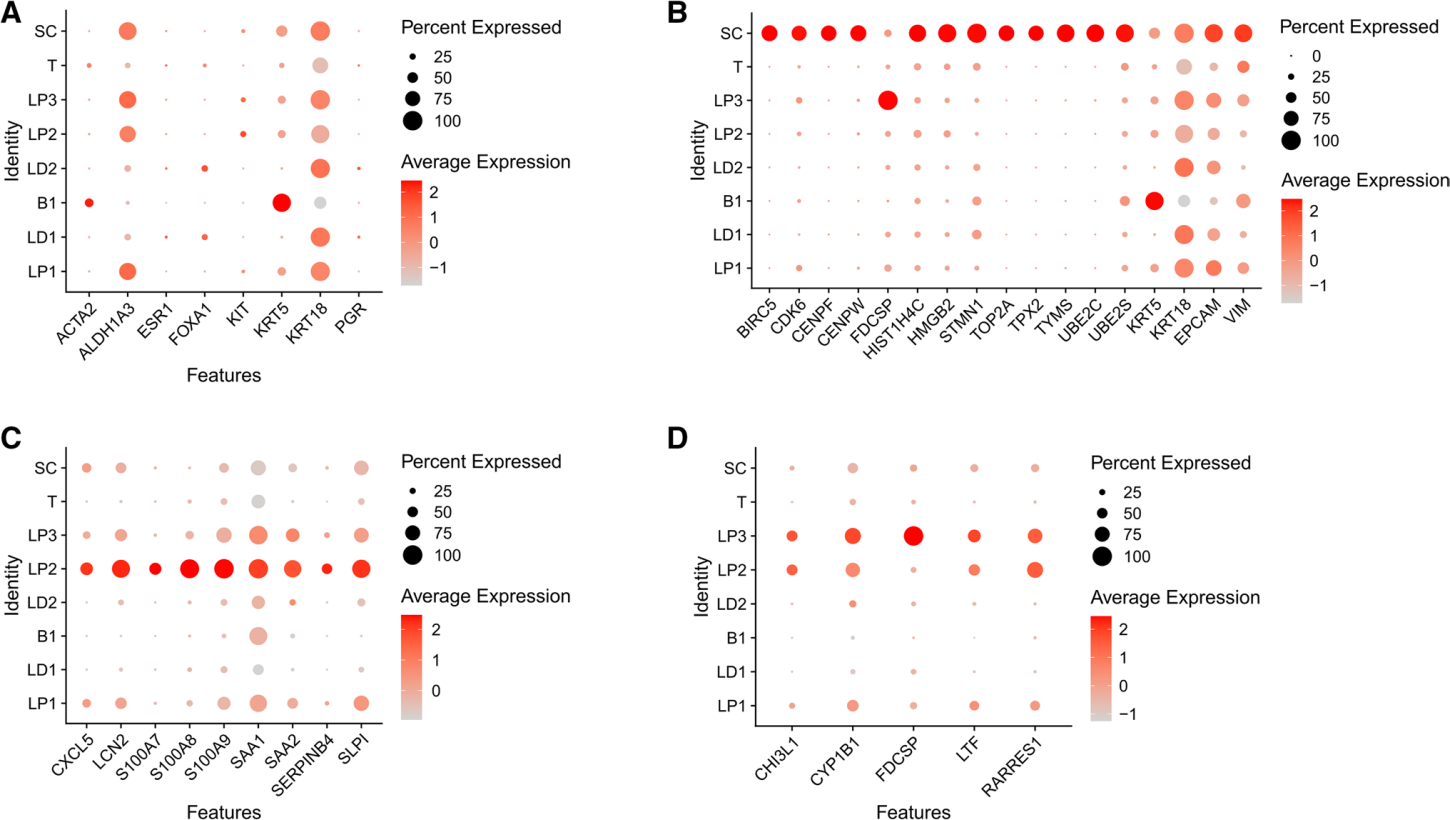
Gene expression in human adult mammary cell populations. Dot plots show the expression of population specific genes in the cell populations at 0.14 cluster resolution. **A** Mammary luminal progenitor, luminal differentiated, and basal cell marker expression in human adult mammary cell populations. **B** SC gene expression in human mammary cells, as well as a basal (KRT5), luminal (KRT18), epithelial (EPCAM), and mesenchymal (VIM) marker expression. **C** LP2 and **D** LP3 marker gene expression in human mammary cells

Figure 2 A shows the expression of the marker genes from Fig. 1D in the gene clusters in a dot plot. The dot plot shows the percentage of cells within a population that express the gene, based on the dot size, and the relative gene expression level, indicated by the dot color. We identified a transitionary population (T) positioned between the luminal and basal cells with low luminal and basal marker expression (Fig. 2 A). As expected, the rest of the markers are localized to the respective cell subpopulations. We also identified the “SC” population as a stem cell-like population because it expresses multiple stem cell-related genes, including ALDH1A3, BIRC5, CDK6, HMGB2, and STMN1 (Fig. 1D and E and 2B). Interestingly, ALDH1A3 identifies mammary luminal progenitor and stem cells [23, 29]. BIRC5 is an oncofetal gene highly expressed in embryonic stem cells [30]. CDK6 regulates exit from quiescence and is required for normal function in hematopoietic stem cells and maintains self-renewal in leukemia stem cells [31–33]. HMGB2 is expressed in proliferating neural stem cells, regulates telomerase activity, and maintains stemness in hematopoietic and mesenchymal stem cells [34–37]. STMN1 is also associated with cycling stem cells [38]. Because of the expression of these genes in “SC,” we identified SC as a stem cell-like population. Genes that were highly expressed in SC were placed in Table 1. In Fig. 2B, we show the cluster-specific expression of the SC genes (Table 1) as well as a luminal (KRT18), basal (KRT5), epithelial (EPCAM), and mesenchymal (VIM) marker gene. Interestingly, SC has luminal, basal, epithelial, and mesenchymal properties based on gene expression. We also show the expression of LP2 and LP3 marker genes and recorded these genes in Table 1 (Fig. 2 C and D).

**Table 1:** Genes highly expressed in the SC, LP2, and LP3 populations, and their expression in mouse mammary populations and human breast cancer

Gene marker	Identified human cell populations	Identified mouse cell populations	GENT2 data- BrC subtype with highest gene expression	GENT2 data- highest Log2 fold change between BrC subtypes
**SC genes**				
BIRC5	SC	Very hi in fMaSC, med in AD1, AP1, B1	Basal	1.994***
CDK6	SC	Medium in all LP, AP, AD, B1 B2, B4, and B5; low in fMaSC	Basal	1.831***
CENPF	SC	AD1, AP1, B1; low in fMaSC	Basal	1.858***
CENPW	SC	Medium in fMaSC, AD1, AP1, B1, low in rest	Basal	2.248***
FDCSP	Hi in LP3, low in rest	–	Basal	4.458***
HIST1H4C	SC, low in rest	Low in fMaSC	Triple-negative	3.214***
HMGB2	SC, low in rest	Very hi in fMaSC, AP1, and AD1, medium in rest	Triple-negative (second highest in Basal)	1.833***
STMN1	Hi in SC, low in rest	Very hi in fMaSC, hi in AP1, AD1, AP2, B1, low in rest	Triple-negative (second highest in Basal)	2.208***
TOP2A	SC	Hi in fMaSC, medium in AP1, AD1, B1	Basal	1.634***
TPX2	SC	fMaSC, AP1, AD1, B1	Basal	2.264***
TYMS	SC	Hi in fMaSC medium in AP1, AD1, B1, low in rest	Basal	1.899***
UBE2C	SC	Very hi in fMaSC, hi in AP1, AD1, B1	Basal	1.949***
UBE2S	Hi in SC, low in rest	Medium-low in all adult cell populations	Basal	1.988***
**LP2 genes**				
CXCL5	LP2	Hi in LP2, medium in LP1 and LP4	Triple-negative	2.142***
LCN2	Hi in LP2, low in rest of LP	Very hi in LP2, medium-low in rest of adult cell populations	Basal	2.587***
S100A7	LP2	Low in LP1 & B3	HER2	5.443***
S100A8	LP2	LP1, LP2	HER2	4.348***
S100A9	Hi in LP2, low in rest of LP	None	HER2	4.637***
SAA1	Hi in LP2, low in rest of LP, B1	AD2, LP2	Triple-negative	2.659***
SAA2	LP2, low in LP3	AD2	–	–
SERPINB4	LP2	–	Luminal	1.503***
SLPI	Hi in LP2, low in rest of LP and SC	Hi in B2, low in rest of adult cell populations	Basal	2.681***
**LP3 genes**				
CHI3L1	LP2, LP3	–	Basal	2.780***
CYP1B1	LP3, low in LP1 and LP2	–	Triple-negative	1.410***
FDCSP	LP3	–	Basal	4.458***
LTF	LP3, low in LP1 and LP2	Hi in AP2, LP2, medium in LP1, LP3, and LP4	HER2	2.825***
RARRES1	LP2, LP3, low in LP1	None	Basal	3.189***

GENT2 data comes from n = 2164 microarrayed patient samples in the GEO database

*fMaSC* fetal mammary stem cells, *B* basal, *AD* differentiated alveolar cells, *AP* alveolar progenitor cells, *LP* luminal progenitor cells, *LD* differentiated luminal cells, *SC* stem cell, - gene not in dataset

*** p < 0.001

### GENT2 identifies breast cancers associated with normal breast population-specific markers

We utilized the tool GENT2 to identify if the population-specific markers from differential expressed gene analysis were strongly associated with specific BrC subtypes. GENT2 runs statistical analysis on a compilation of microarray data from 2,164 BrC patient tumor samples [25]. We recorded the resulting analyses in Table 1. Table 1 shows the BrC subtype that most expresses the gene marker and the largest log 2-fold gene expression difference between subtypes. Significant fold change differences between BrC subtypes indicate that the cell population marker is also BrC subtype-specific and can be a promising marker for BrC diagnosis. LD2, T, and B1 related genes are not strongly associated with specific human BrC subtypes or generally have low log 2-fold change values in the GENT2 database (Table S2). LP1 marker genes had considerable expression overlap with the other LP populations and SC, and LD1 had considerable overlap with LD2, so we did not look into these or the above populations further (Table S2).

The best LP3 marker gene, FDCSP, is highly expressed specifically in Basal-like BrC, and the less specific LP3 marker genes are also highly expressed in Basal-like BrC (Table 1). However, the LP3 marker genes are not all expressed primarily in one cancer subtype, and this suggests that it either does not give rise to cancer subtypes or can give rise to several cancer subtypes. Interestingly, in LP2, the most specific marker genes, S100A7, S100A8, and S100A9, are explicitly expressed in HER2 BrCs in the GENT2 database (Table 1). We hypothesize that LP2 may be the cell population that becomes HER2-overexpressing BrC.

### Expression of human population-specific marker genes in mouse mammary gland cell populations

**Fig. 3:**
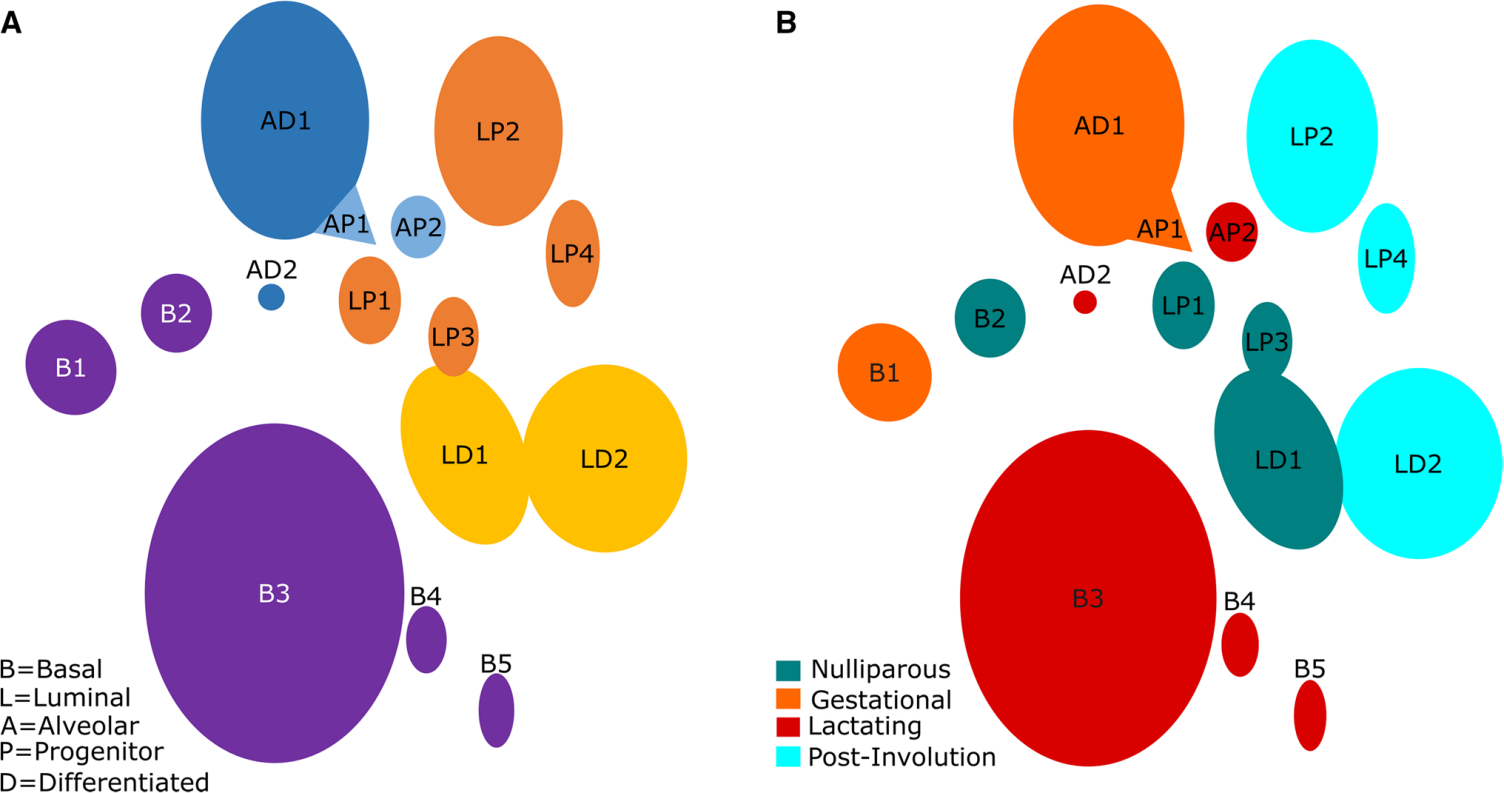
Mammary cell populations in the adult mouse in nulliparous, gestational, lactating, and post-involution stages.** A** 15 cell populations, basal (purple), differentiated alveolar (blue), alveolar progenitor (light blue), luminal progenitor (orange), and luminal differentiated (yellow) cell populations are shown. **B** 15 cell populations, nulliparous (teal), gestational (orange), lactating (red), and post- involution (turquoise) cell populations are shown. Based on the work of Bach et al. [9]

Having extracted many exciting data from this human dataset, we then examined mouse mammary RNAseq/scRNAseq datasets using graphical user interfaces designed by the data’s creators [9, 10]. As stated in the introduction, the mouse datasets are more fleshed out in terms of cell populations in mammary developmental stages than the current human datasets, having fetal cells and adult nulliparous, gestational, lactating, and post-involution cells. We thought these datasets could contribute to our study. In the mouse datasets, there are fetal mammary stem cells (fMaSC), five basal cell populations (named by us B1–B5), two differentiated alveolar cell populations (named by us AD1-2), two alveolar progenitor cell populations (named by us AP1-2), four luminal progenitor cell populations (named by us LP1-4), and two differentiated luminal cell populations (named by us LD1-2). These cell populations and some identifying markers were established by the original authors of the data [9, 10, 12]. Figure 3A shows a representation of the adult mouse mammary epithelial cell populations based on the work of Bach and colleagues [9]. Figure 3B shows the mouse mammary cell populations in the nulliparous, gestation, lactation, and post involution states. We identified the expression of the Table 1 genes in the mouse datasets, determined which mouse cell population expressed the gene, and recorded these populations in Table 1. We found some exciting results.

In the mouse data, the majority of the specific markers for the human SC population (9 of 12), BIRC5, CENPF, CENPW, HMGB2, STMN1, TOP2A, TPX2, TYMS, and UBE2C, are expressed in gestational-specific mouse cell populations AP1, AD1, and B1 (Fig. 3B; Table 1) [9]. Further, most of the SC genes (11 of 12) were expressed at some level, specifically in fMaSCs (Table 1), reinforcing our theory that SC is indeed a mammary stem cell population [10]. The majority of the SC population is from tissue sample PM0372-Epi, a parous postmenopausal woman. Further, in the GENT2 data, most of these markers are highly expressed primarily in basal-like BrCs, and two of the three exceptions, HMGB2 and STMN1, have the second-highest expression in basal-like BrCs. We hypothesize that SC is a remnant of a human gestational or fetal cell population. Further, we hypothesize that SC is the cell of origin for many basal-like BrCs.

### Expression of normal breast stem cells markers in scRNAseq TN human breast cancer

**Fig. 4:**
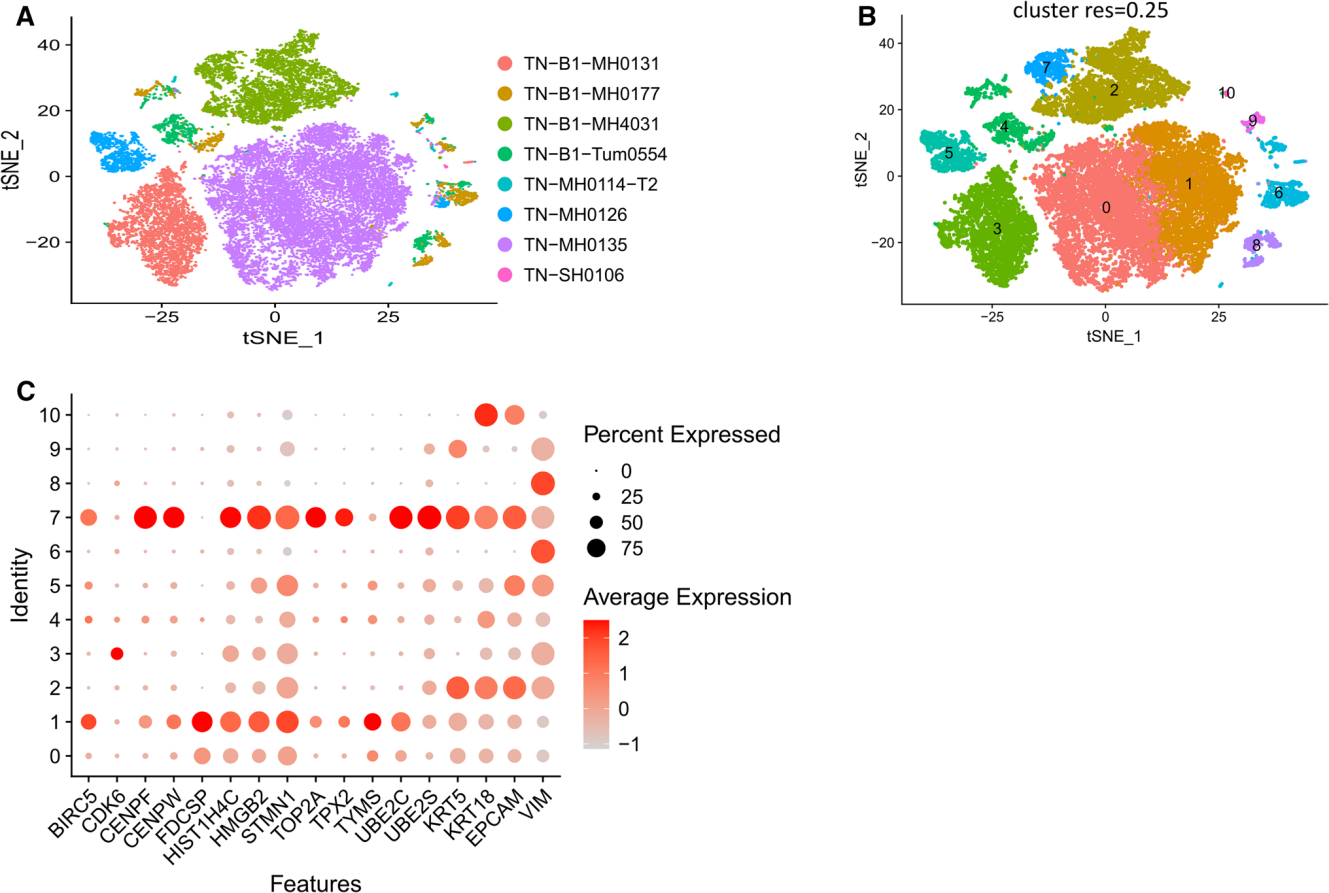
SC gene expression in eight human triple-negative breast cancer cell datasets.** A** t-SNE plot of eight triple-negative breast scRNA-seq datasets. Individual dots correspond to cells and dot color indicates individual tissue sample. **B** The cells from the t-SNE plot of A was broken down into eleven cell clusters at 0.25 cluster resolution based on shared gene expression patterns. The dot color and number represent cell clusters. **C** Dot plot showing the expression of SC genes in the breast cancer cell populations, as well as a basal (KRT5), luminal (KRT18), epithelial (EPCAM), and mesenchymal (VIM) marker

Having examined SC gene expression in GENT2 BrC data and in mouse datasets, we then examined eight TN BrC scRNAseq datasets from GSE161529. Figure 4A shows each patient’s cells (dots) in a t-SNE plot. The dot color indicates the patient sample. Notably, most TN BrCs formed individual non-overlapping groups, demonstrating the variability within the TN subtype. Figure 4B shows the cells from Fig. 4A clustered based on shared gene expression patterns at a 0.25 resolution. The dot color now indicates which cluster patient cells belong to. We then examined the SC marker genes in these clusters. The resultant dot plot shows that two cancer cell populations highly express many SC genes (Fig. 4C). Populations 1 and 7 have high SC gene expression. Notably, population 7 is from a BRCA mutant dataset while 1 is BRCA normal. Based on the SC gene expression, it appears that populations 1 and 7 are potential cancer stem cell (CSC) populations. Populations 1 and 7 both have adjacent cell populations, 0 and 2, from the same respective patient samples. These last two cell populations are likely composed of cells that differentiated from the respective CSC populations.

### Expression of LP2 genes in an assorted panel of BrC cell lines

**Fig. 5:**
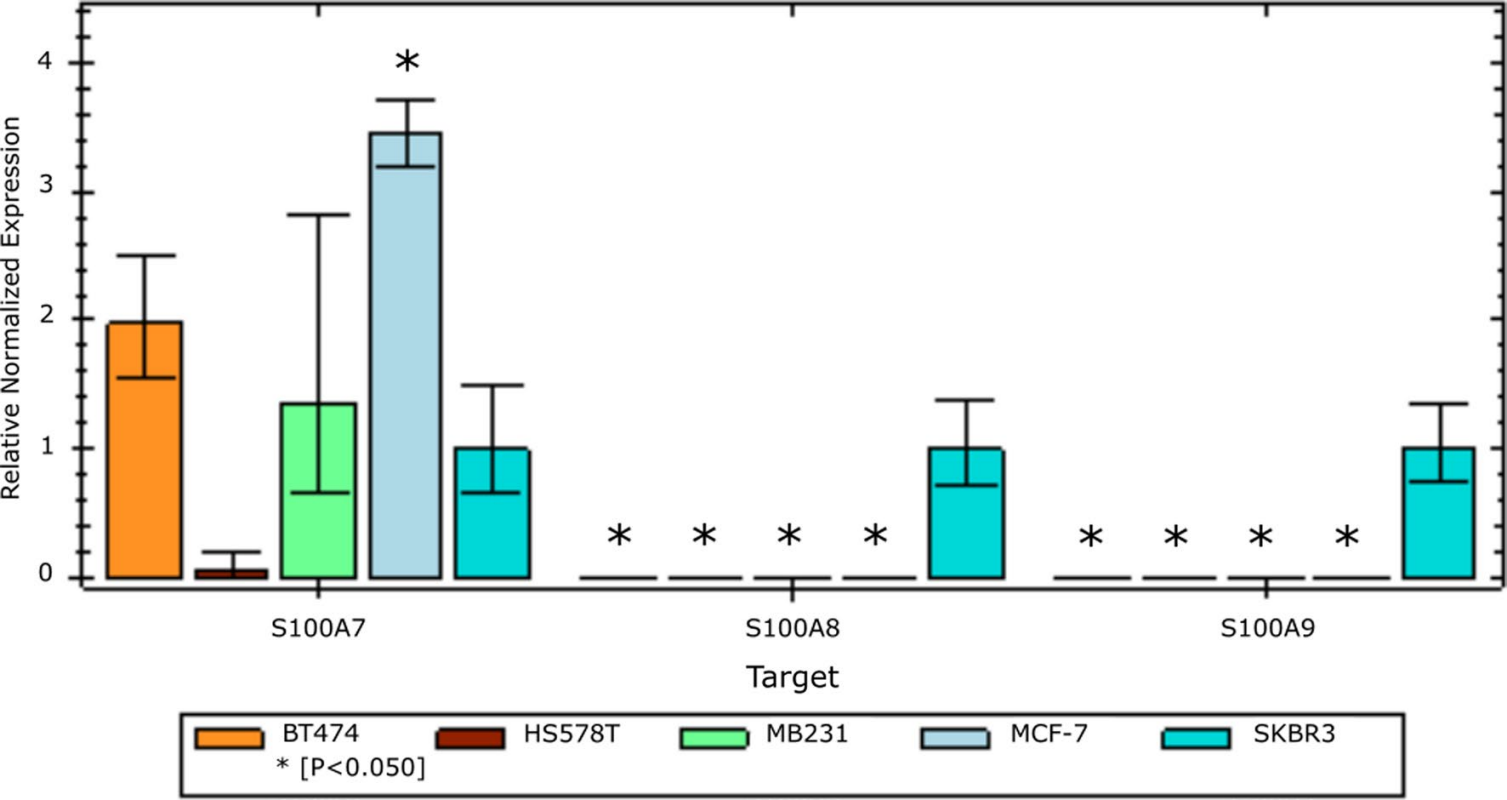
LP2 gene expression in breast cancer cell lines. RT-qPCR bar graphs of relative normalized RNA expression of LP2 genes in BrC cell lines. Gene expression from three LP2 genes (S100A7, A8, A9) were examined in MCF-7 (Luminal A, light blue), BT474 (Luminal B, orange), SKBR3 (HER2-overexpressing, turquoise), HS578T (TN claudin-low, brown), and MB231 (TN claudin-low, green). The error bars show standard error of the mean. Three independent experiments are shown. * indicates p < 0.050 compared to SKBR3

Above we provided evidence of SC marker genes presence in TN BrC cells. We also wanted to provide evidence beyond the GENT2 database for the presence of LP2 markers in the Her2-overexpressing BrC subtypes. So, we obtained a panel of five assorted BrC cell lines and examined normalized relative mRNA expression for the three LP2 genes (S100A7, S100A8, and S100A9). Figure 5 shows bar graphs of the relative normalized RT-qPCR data. Two of the three genes (S100A8 and S100A9) were significantly expressed only in the Her2-overexpressing BrC cell line, SKBR3. The creation of cell lines and the use of adherent culture do not fully mimic the tissue microenvironment or native tissues, so we do not find it surprising that one gene, S100A7, was expressed in other BrC cell lines to a comparable or higher level. Overall, this data bolsters the GENT2 data and shows the overexpression of the majority of the LP2 genes in Her2-overexpressing BrC cell lines.

Overall, this research suggests that a gestation or fetal specific stem-cell population exists in adult human breast tissue and that this population could be the origin of Basal-like BrCs and Basal-like CSCs. Further, the identified marker genes for this population are highly population-specific in the mammary gland, are related to stem cell function, and are, therefore, promising targets for treatment in Basal-like BrC. We also identified a luminal progenitor population marked by S100A7, S100A8, and S100A9 that may be the origin of HER2 enriched BrCs.

## Discussion

The cell populations of origin for specific BrC subtypes are generally unknown. If these specific cell populations are identified, we can identify cellular pathways that contribute to subtype-specific carcinogenesis and potentially screen patients for malignant mutations in these cell populations that would predispose patients to BrC. This research compares normal breast cell populations to human BrC subtypes and normal mouse mammary gland populations. We identified shared gene expression patterns and predicted two cell populations that may transform into Basal-like and HER2-overexpressing BrCs, respectively. Basal BrC is a subtype of TN, and we went on to examine TN BrC scRNAseq datasets [3]. From this research, we identify two potential CSC populations within a subset of TN BrCs. These CSCs highly expressed many of the SC genes.

The identified normal SC population and the corresponding CSC populations have known mammary gland stemness properties. SC and the CSCs have luminal, basal, epithelial, and mesenchymal marker expression. Mammary stem cells are known to express both luminal and basal markers and even vimentin, a mesenchymal marker [29, 39]. Also, partial EMT, which is characterized by co-expression of epithelial and mesenchymal markers, identifies BrC cells with stem cell properties [40, 41].

The SC population we identified expressed twelve marker genes, BIRC5, CDK6, CENPF, CENPW, HIST1H4C, HMGB2, STMN1, TOP2A, TPX2, TYMS, UBE2C, and UBE2S. The scientific literature not only corroborates that many of these genes are stem cell related, but also that they are specifically associated with the basal-like BrC subtype. BIRC5, CENPF, FDCSP, HIST1H4C, HMGB2, STMN1, TYMS, UBE2C, and UBE2S are upregulated in BrCs, specifically in basal-like BrCs for BIRC5, CENPF, STMN1, TYMS [4, 42–55]. In several papers where TN BrCs were not subdivided to include a separate section for the basal-like subtype, the TN subtype showed high expression of SC marker genes. CENPW, TPX2, and UBE2C are overexpressed in TN BrCs [56–58]. UBE2C expression is upregulated in HER2 expressing and TN BrCs [49, 50].

Many of the SC marker genes are correlated with poor BrC patient survival statistics in the literature. BrCs expressing BIRC5, CENPF, or UBE2C have reduced disease-free, metastasis-free, and overall survival [43, 50, 52, 59, 60]. HIST1H4C expression is associated with worse overall and metastasis-free survival in BrCs [46]. HMGB2, STMN1, and TPX2 expression is correlated with worse disease-free and overall survival [47, 54, 58, 61, 62]. TYMS expressing BrCs have low overall survival [48].

Several SC marker genes have known stem cell-related properties in BrCs. STMN1 expression is associated with the CD44+/CD24- BrC stem cell phenotype [61]. TYMS maintains BrC spheroid formation efficiency and CD24- status [48]. TPX2 and UBE2C knockdown reduce colony formation efficiency in TN BrCs [57]. Lastly, UBE2S knockdown suppresses anchorage-independent growth in BrCs [51].

Interestingly, several breast cancer papers identify co-expression of the SC marker genes. CENPF expression correlates with BIRC5 expression [4, 60]. TPX2 and UBE2C were highly expressed in the same TN BrC cell populations and cell lines [57]. Overall, the scientific literature corroborates and greatly bolsters the association of the SC markers with basal-like BrC.

Most SC marker genes are specifically upregulated in basal-like or TN BrC subtypes, and we hypothesize that malignant SC cells create basal-like BrCs. These marker genes were almost exclusively in the SC population in the normal human breast epithelium; therefore, these genes could be promising targets for targeted treatment of basal-like BrC with minimal local normal tissue damage. For instance, the SC gene BIRC5 is an onco-fetal protein rarely expressed in adult tissues and BIRC5 inhibitors have been shown to be effective in *in vitro* BrC treatment [63, 64]. A CENPF inhibitor also shows promise in BrC treatment *in vitro* [65].

Besides the SC population, SC marker genes, and potential TN CSC populations, we also identified a S100A7, S100A8, and S100A9 expressing normal mammary luminal progenitor cell population. We determined that these three genes are strongly associated with the HER2 BrC subtype and hypothesized that transformed cells from this population become HER2-overexpressing BrCs. Many scientific papers have shown that these three genes are associated with HER2 BrCs and are often correlated with poor BrC patient outcomes. S100A7 expression is negatively correlated with ESR1 and PGR in human BrCs and correlated with decreased disease-free and overall survival [66, 67]. High S100A8 expression is positively correlated with HER2 expression and negatively correlated with ESR1 and PGR expression [68–70]. Further, high S100A8 expression is associated with increased cancer relapse and lower overall and disease-free survival [68]. S100A9 has also been correlated with HER2 BrCs and poor overall survival [69–71]. Further, a Luminal A cell line treated with S100A8/A9 had a marked decrease in ESR1 expression, suggesting that S100A8/A9 may have a causal role in the HER2 BrC phenotype [69]. Together, there is strong evidence in the literature that S100A7, S100A8, and S100A9 are negative BrC prognostic markers and are associated with HER2 BrCs, as we suggest in this research.

Interestingly, S100A7, S100A8, and S100A9 are also associated with stem-cell properties in BrC. In BrC, expression of these three genes is associated with effective mammosphere formation and inhibition of these genes stunted mammosphere growth and xenograft tumor growth [72].

As with the SC marker genes, S100A7, S100A8, and S100A9 are promising targets for treatment. We found that these genes are almost exclusively in one luminal progenitor cell population, suggesting that targeted treatment for these genes in HER2 BrC would not cause significant off-target damage to most breast cells, contributing to better patient outcomes.

## Conclusions

In conclusion, we identified a normal stem cell-like cell population and its marker genes in adult human breast tissue. The marker genes are expressed almost exclusively in gestation-specific adult mouse cell populations, suggesting that it is a human gestation-specific stem cell population. Further, many of these genes are specifically expressed in basal-like and TN BrC. We identified two potential CSC populations within a subset of TN BrCs. These populations highly express many of the SC genes and may represent normal stem cells that have become cancerous. We also identified a novel normal luminal progenitor cell population marked by three genes (S100A7, S100A8, S100A9) that are also explicitly overexpressed in HER2 breast cancer. We hypothesize that these two cell populations give rise to the basal-like/TN and HER2-overexpressing breast cancer subtypes, respectively.

## Supplementary Information

Supplementary material 1 (PDF 246.2 kb)

## Data Availability

Publicly available datasets were used for this research. The GSE161529 dataset was produced by Pal and colleges and is available in the Gene Expression Omnibus (GEO) archive [7]. GENT2 data was from the GENT2 server at http://gent2.appex.kr/gent2/ [25]. https://wahl-lab-salk.shinyapps.io/Mammary_snATAC and https://marionilab.cruk.cam.ac.uk/mammaryGland are the website addresses that display the mouse datasets in a user-friendly research format provided by their respective authors.

## Code availability

The workflows we used with R are stored as supplementary word files.
