# Supplementary material for: Newly identified breast luminal progenitor and gestational stem cell populations likely give rise to HER2-overexpressing and basal-like breast cancers
Source: Discov Oncol. 2022 May 28;13:38. doi: 10.1007/s12672-022-00500-6 (PMC9148339; doi:10.1007/s12672-022-00500-6)
Supplement: Supplementary file 1 — Supplementary material 1 (PDF 246.2 kb) [file 12672_2022_500_MOESM1_ESM.pdf]

Additional File

Newly identified breast luminal progenitor and gestational stem cell populations likely give rise to HER2-overexpressing and basal-like breast cancers.

James R. W. McMullen[a], Ubaldo Soto[a]

[a] Department of Basic Sciences, School of Medicine, Loma Linda University, Loma Linda, CA 92350, USA

Correspondence: Ubaldo Soto, Ph.D., Loma Linda University School of Medicine, 11021 Campus Street, Alumni Hall, Room 117, Loma Linda, California 92350, United States, Telephone: +1-909-558-7562 |fax: +1-909-558-4035, email: usoto@llu.edu

Supplementary Table S1. Primers used for RT-qPCR

Gene Name	Forward Primer	Reverse Primer
28S rRNA	CAATGAAGCGCGGGTAAACG	ATGTCTCTTCACCGTGCCAG
ACTB (Beta Actin)	GCACAGAGCCTCGCCTTT	TATCATCATCCATGGTGAGCTGG
B2M	AAGATGAGTATGCCTGCCGT	CTGCTTACATGTCTCGATCCCA
GAPDH	CTCCTCCACCTTTGACGCT	GCCAAATTCGTTGTCATACCAGG
S100A7	TGGGAGACATAGCCACAGAC	GACATTTTATTGTTCCTGGGGTCTC
S100A8	AAGGGGAATTTCCATGCCGT	ACGTCTGCACCCTTTTTCCT
S100A9	CTCCTCGGCTTTGACAGAGT	TGGTCTCTATGTTGCGTTCCA

Supplementary Table S2. Genes highly expressed in the LD1, LD2, LP1, T, and B1 populations, and their expression in human breast cancer.

Gene Marker	Identified Human Cell Populations	GENT2 data- BrC subtype with highest gene expression	GENT2 data- highest Log2 fold change between BrC subtypes
**LD1 genes**			
**AREG**	LD1. LD2, T	Luminal	2.337***
**AZPG1**	LD1, LD2	Luminal A	2.678***
**CST3**	LD1, LD2	Luminal	2.673***
**HSPB1**	LD1, LD2, T	Luminal B	2.021***
**STC1**	LD1	Luminal	0.880***
**STC2**	LD1, LD2	Luminal A	2.989***
**SOX4**	LD1, LD2, T	Triple-negative	1.291***
**TCIM**	LD1, LD2	-	-
**TFF1**	LD1, LD2	Luminal B	5.563***
**TFF3**	LD1, LD2	Luminal B	4.664***
**LD2 genes**			
**AGR2**	LD2	Luminal B	6.068***
**APOD**	LD2	Luminal A	2.120***
**HMGB3**	LD2	Triple-negative	1.507***
**MUCL1**	LD2	HER2	5.290***
**PIP**	LD2	Luminal A	4.700***
**SERPINA1**	LD2	Luminal	2.019***
**TFPI2**	LD2	Triple-negative	0.501*
**LP1 genes**			
**ALDH1A3**	LP1-3, SC	Triple-negative	1.659***
**ANXA1**	LP1-3, SC, T, B1	Triple-negative	1.725***
**CTSV**	LP1, LP3, SC	Basal	2.003***
**HMGA1**	LP1-3, SC	Basal	1.254***
**KRT15**	LP1-3, SC	Basal	2.022***
**KRT16**	LP1-3, SC	Basal	4.199***

**KRT81**	LP1-3, SC	Basal	2.856***
**MMP7**	LP1-3, SC	Triple-negative	2.731***
**PI3**	LP1, LP2	Basal	2.194***
**SCGB3A1**	LP1, LP3, LD2, SC	Luminal A	1.632***
**T genes**			
**CXCL2**	T, low in B1	Basal	1.576***
**DNAJB1**	T	Luminal	0.672***
**EGR1**	T	Luminal A	1.760***
**FOS**	T	Luminal A	2.378***
**GADD45B**	T, low in LD1 and LD2	Luminal A	0.380***
**HSPA6**	T	Luminal	1.092***
**HSPA1A**	T	Luminal B	0.502***
**HSPA1B**	T	Triple-negative	0.895***
**JUN**	T, low in rest	Luminal A	0.454***
**MT1A**	T	-	-
**B1 genes**			
**ACTG2**	B1	Basal	1.769***
**CAV1**	B1	Triple-negative	1.048***
**CRYAB**	B1, T	Basal	2.437***
**DST**	B1	Luminal A	0.408***
**KRT5**	B1	Basal	3.586***
**KRT14**	B1	Basal	2.345***
**KRT17**	B1	Basal	3.079***
**NNMT**	B1	Triple-negative	1.162*
**TAGLN**	B1	Luminal A	1.082***
**TPM2**	B1	Luminal A	0.861***

*** $p \leq 0.05$, *** $p < 0.001$**
GENT2 data comes from n=2164 microarrayed patient samples from the GEO database
T transitionary, B basal, LP luminal progenitor, LD differentiated luminal cells, SC stem-cell like cells

Code Used to examine normal breast cell scRNAseq data in R

Partial install list:

```
BiocManager::install("glmGamPoi")
BiocManager::install("GO.db")
BiocManager::install("org.Hs.eg.db")
devtools::install_github('xzhoulab/iDEA')
install.packages("sctransform")
install_github("BaderLab/scClustViz")
install.packages("BH")
install_github("immunogenomics/presto")
install.packages("limma")
```

Start Seurat:

```
	library(Seurat)
	library(dplyr)
library(ggplot2)
library(cowplot)
library(Matrix)
library(Biobase)
library(RColorBrewer)
library(data.table)
library(ggthemes)
library(devtools)
library(BH)
library(scClustViz)
library(presto)
library(sctransform)
library(glmGamPoi)
library(limma)
library(GO.db)
library(org.Hs.eg.db)
library(iDEA)
```

```
samples = c("N1105-Epi","N280-Epi","MH0023-Epi","MH0064-Epi","PM0342-Epi","PM0372-Epi","MH275-Epi")
outdir = "/users/jmcmullen/Downloads/GSE161529";

memory.limit(size=200000)
```

```
data.10x = list();
data.10x[[1]] <- Read10X(data.dir
='/users/jmcmullen/Downloads/GSE161529/GSM4909260_N-N1105-Epi');
data.10x[[2]] <- Read10X(data.dir
='/users/jmcmullen/Downloads/GSE161529/GSM4909255_N-N280-Epi');
data.10x[[3]] <- Read10X(data.dir
='/users/jmcmullen/Downloads/GSE161529/GSM4909267_N-MH0023-Epi');
data.10x[[4]] <- Read10X(data.dir
='/users/jmcmullen/Downloads/GSE161529/GSM4909262_N-MH0064-Epi');
data.10x[[5]] <- Read10X(data.dir
='/users/jmcmullen/Downloads/GSE161529/GSM4909269_N-PM0342-Epi');
data.10x[[6]] <- Read10X(data.dir
='/users/jmcmullen/Downloads/GSE161529/GSM4909275_N-PM0372-Epi');
data.10x[[7]] <- Read10X(data.dir
='/users/jmcmullen/Downloads/GSE161529/GSM4909273_N-MH275-Epi');

scrna.list = list();
##can remove or change filtering parameters (min.cells=100, min.features=500,)
for (i in 1:length(data.10x)) {scrna.list[[i]] = CreateSeuratObject(counts = data.10x[[i]],
min.cells=100, min.features=500, project=samples[i]); scrna.list[[i]][["DataSet"]] = samples[i];}

rm(data.10x);

scrna <- merge(x=scrna.list[[1]],
y=c(scrna.list[[2]],scrna.list[[3]],scrna.list[[4]],scrna.list[[5]],scrna.list[[6]],scrna.list[[7]]),
add.cell.ids = c("1","2","3","4","5","6","7"), project="VisvaderN")

rm(scrna.list);

str(scrna@meta.data)



saveRDS(scrna, file = sprintf("%s/Vis2021_7N_12-16.rds", outdir));

##QC procedures_1

scrna[[]];
str(scrna@meta.data);

View(scrna)

mito.genes <- grep(pattern = "^MT-", x = rownames(x = scrna), value = TRUE);
percent.mito <- Matrix::colSums(x = GetAssayData(object = scrna, slot = 'counts')[mito.genes, ])
/ Matrix::colSums(x = GetAssayData(object = scrna, slot = 'counts'));
scrna[['percent.mito']] <- percent.mito;
```

```
ribo.genes <- grep(pattern = "^RP[SL][[:digit:]]", x = rownames(x = scrna), value = TRUE);
percent.ribo <- Matrix::colSums(x = GetAssayData(object = scrna, slot = 'counts')[ribo.genes, ])
/ Matrix::colSums(x = GetAssayData(object = scrna, slot = 'counts'));
scrna[['percent.ribo']] <- percent.ribo;

pdf(sprintf("%s/Scatter1.pdf", outdir), width = 8, height = 6);
scatter <- FeatureScatter(object = scrna, feature1 = "nCount_RNA", feature2 = "percent.mito",
pt.size=0.1)
print(scatter);
dev.off();
pdf(sprintf("%s/VlnPlot.pdf", outdir), width = 13, height = 6);
vln <- VlnPlot(object = scrna, features = c("percent.mito", "percent.ribo"), pt.size=0, ncol = 2,
group.by="DataSet");
print(vln);
dev.off();
pdf(sprintf("%s/VlnPlot.nCount.25Kmax.pdf", outdir), width = 10, height = 10)
vln <- VlnPlot(object = scrna, features = "nCount_RNA", pt.size=0, group.by="DataSet",
y.max=25000)
print(vln)
dev.off();
pdf(sprintf("%s/VlnPlot.nFeature.pdf", outdir), width = 10, height = 10)
vln <- VlnPlot(object = scrna, features = "nFeature_RNA", pt.size=0, group.by="DataSet")
print(vln)
dev.off()
pdf(sprintf("%s/Scatter2.pdf", outdir), width = 8, height = 6);
scatter <- FeatureScatter(object = scrna, feature1 = "nCount_RNA", feature2 = "percent.ribo",
pt.size=0.1)
print(scatter);
dev.off();
pdf(sprintf("%s/Scatter3.pdf", outdir), width = 8, height = 6);
scatter <- FeatureScatter(object = scrna, feature1 = "nCount_RNA", feature2 = "nFeature_RNA",
pt.size=0.1)
print(scatter);
dev.off();

min <- min(scrna@meta.data$nFeature_RNA);
m <- median(scrna@meta.data$nFeature_RNA)
max <- max(scrna@meta.data$nFeature_RNA)
s <- sd(scrna@meta.data$nFeature_RNA)
min1 <- min(scrna@meta.data$nCount_RNA)
max1 <- max(scrna@meta.data$nCount_RNA)
m1 <- mean(scrna@meta.data$nCount_RNA)
s1 <- sd(scrna@meta.data$nCount_RNA)
Count93 <- quantile(scrna@meta.data$nCount_RNA, 0.93) # calculate value in the 93rd
percentile
```

```
print(paste("Feature stats:",min,m,max,s));
print(paste("UMI stats:",min1,m1,max1,s1,Count93));

scrna <- subset(x = scrna, subset = nFeature_RNA > 500  & percent.mito < 0.2)

##QC procedures_2
View(scrna@meta.data)
#nfeatures_RNA is the number of genes /cell
#nCount_RNA is the number of unique molecular identifier (UMI) / cell
scrna$log10GenesPerUMI <- log10(scrna$nFeature_RNA) / log10(scrna$nCount_RNA)
#above command creates a ratio between nfeatures/nCount in the metadata

scrna$mitoRatio <- PercentageFeatureSet(object = scrna, pattern = "^MT-")

#extract metadata frame to modify w/o messing up existing metadata
metadata <- scrna@meta.data

metadata <- metadata %>% dplyr::rename(seq_folder = orig.ident, nUMI = nCount_RNA,
nGene = nFeature_RNA)
#change names of features

View(metadata)

scrna@meta.data <- metadata


#visualize number of cells per sample
metadata %>% ggplot(aes(x= seq_folder, fill= seq_folder)) + geom_bar() + theme_classic() +
theme(axis.text.x = element_text(angle = 45, vjust = 1, hjust=1)) + theme(plot.title =
element_text(hjust=0.5, face="bold")) + ggtitle("NCells")

#visualize UMI #/cell
metadata %>% ggplot(aes(color=seq_folder, x=nUMI, fill=seq_folder)) + geom_density(alpha =
0.2) + scale_x_log10() + theme_classic() + ylab("Cell density") + geom_vline(xintercept =
2000)
# number of genes/cell
metadata %>% ggplot(aes(color=seq_folder, x=nGene, fill=seq_folder)) + geom_density(alpha =
0.2) + theme_classic() + scale_x_log10() + geom_vline(xintercept = 800)

# Cell number vs gene number
metadata %>% ggplot(aes(x=seq_folder, y=log10(nGene), fill=seq_folder)) + geom_boxplot() +
theme_classic() + theme(axis.text.x = element_text(angle = 45, vjust = 1, hjust=1)) +
theme(plot.title = element_text(hjust=0.5, face="bold")) + ggtitle("NCells vs NGenes")

# number genes vs number UMI with high mtgene identified
```

```
metadata %>% ggplot(aes(x=nUMI, y=nGene, color=percent.mito)) + geom_point() +
scale_colour_gradient(low = "gray90", high = "black") + stat_smooth(method=lm) +
scale_x_log10() + scale_y_log10() + theme_classic() + geom_vline(xintercept = 500) +
geom_hline(yintercept = 250) + facet_wrap(~seq_folder)

# mtgene / cell
metadata %>% ggplot(aes(color=seq_folder, x=percent.mito, fill=seq_folder)) +
geom_density(alpha = 0.2) + scale_x_log10() + theme_classic() + geom_vline(xintercept = 0.2)

#genes/UMI to visualize complexity
metadata %>% ggplot(aes(x=log10GenesPerUMI, color =seq_folder, fill=seq_folder)) +
geom_density(alpha = 0.2) + theme_classic() + geom_vline(xintercept = 0.8)

##filtering out cells based on cutoffs
scrna <- subset(x = scrna, subset= (nUMI >= 1500) & (nGene >= 500) & (log10GenesPerUMI >
0.80) & (percent.mito < 0.20))

##filtering out genes not expressed in any cells- extract counts metric, examine number of cells
##expressing gene and make cutoffs, (10+ cells in this example)
counts <- GetAssayData(object = scrna, slot = "counts")
nonzero <- counts > 0
keep_genes <- Matrix::rowSums(nonzero) >= 10
filtered_counts <- counts[keep_genes, ]
scrna <- CreateSeuratObject(filtered_counts, meta.data = scrna@meta.data)

saveRDS(scrna, file = sprintf("%s/Vis2021_7N_12-16_filter.rds", outdir));

##End QC procedures



scrna <- PercentageFeatureSet(scrna, pattern = "^MT-", col.name = "percent.mt")
scrna <- SCTransform(scrna, method = "glmGamPoi", vars.to.regress = "percent.mt", verbose =
TRUE)

scrna <- RunPCA(object = scrna, npcs = 100, verbose = FALSE);
scrna <- ProjectDim(object = scrna)

pdf(sprintf("%s/VizDimLoadings.pdf", outdir), width = 8, height = 30);
vdl <- VizDimLoadings(object = scrna, dims = 1:3)
print(vdl);
dev.off();

pdf(sprintf("%s/PCA.heatmap.multi.pdf", outdir), width = 8.5, height = 24);
hm.multi <- DimHeatmap(object = scrna, dims = 1:12, cells = 500, balanced = TRUE);
print(hm.multi);
```

```
dev.off();

elbow <- ElbowPlot(object = scrna, ndims = 100)
pdf(sprintf("%s/PCA.elbow.pdf", outdir), width = 6, height = 8);
print(elbow);
dev.off();

saveRDS(scrna, file = sprintf("%s/Vis2021_7N_12-16_filter_PCA.rds", outdir));



nPC = 100;
scrna <- RunUMAP(object = scrna, reduction = "pca", dims = 1:nPC);
scrna <- RunTSNE(object = scrna, reduction = "pca", dims = 1:nPC);
scrna.list <- SplitObject(scrna, split.by = "orig.ident")

for (i in 1:length(scrna.list)) {scrna.list[[i]] <- PercentageFeatureSet(scrna.list[[i]], pattern =
"^MT-", col.name = "percent.mt")
scrna.list[[i]] <- SCTransform(scrna.list[[i]], method = "glmGamPoi", vars.to.regress =
"percent.mt", verbose = TRUE)}

anchors <- FindIntegrationAnchors(object.list = scrna.list, dims = 1:100)
scrna.integ <- IntegrateData(anchorset = anchors, dims = 1:100)

DefaultAssay(scrna.integ) <- "integrated" #"integrated" and "RNA" are stored

scrna <- scrna.integ


scrna <- PercentageFeatureSet(scrna, pattern = "^MT-", col.name = "percent.mt")
scrna <- SCTransform(scrna, method = "glmGamPoi", vars.to.regress = "percent.mt", verbose =
TRUE)


saveRDS(scrna, file = sprintf("%s/Vis2021_7N_12-16_filter_anchor.rds", outdir));


scrna <- RunPCA(object = scrna, npcs = 100, verbose = FALSE);
scrna <- ProjectDim(object = scrna)

nPC = 100;
scrna <- RunUMAP(object = scrna, reduction = "pca", dims = 1:nPC);
scrna <- RunTSNE(object = scrna, reduction = "pca", dims = 1:nPC);

pdf(sprintf("%s/tSNE.%d.pdf", outdir, nPC), width = 10, height = 8);
p1 <- DimPlot(object = scrna, reduction = "tsne", group.by = "DataSet", pt.size=0.1)
```

```
print(plot_grid(p1));
dev.off();


nPC = 100;
#0.015 resolution used by Pal et al
scrna <- FindNeighbors(object = scrna, dims = 1:nPC);
scrna <- FindClusters(object = scrna, resolution = c(0.05, 0.1, 0.11, 0.13, 0.15, 0.2));

#test multiple cluster.res values (gen 0.2 – 1.2) and run findneighbor/find cluster

DimPlot(scrna, reduction = "tsne", label = TRUE) + NoLegend() #make a simple plot of clusters
#use above plots to identify good looking clusters

#remove columns for cluster.res values that make bad looking plots
scrna$SCT_snn_res.0.6 <- NULL

#rename clusters based on gene expression
scrna@active.ident <- plyr::mapvalues(scrna@active.ident, c(0, 1, 2, 3, 4, 5, 6, 7), c("LP1",
"LD1", "B1", "LD2", "LP2", "LP3", "T", "SC"))
DimPlot(object = scrna, reduction = "tsne", label.size = 5, pt.size = 0.75)


#remove cluster(s) from analysis
scrna <- subset(scrna, idents = 8, invert = TRUE)


cluster.breakdown <- table(scrna@meta.data$DataSet, scrna@meta.data$seurat_clusters);

DEGs <- FindAllMarkers(object=scrna, logfc.threshold=1, min.diff.pct=.2);
write.table(DEGs, file=sprintf("%s/DEGs.Wilcox.xls", outdir), quote=FALSE, sep="\t",
row.names=FALSE);

top10 <- DEGs %>% group_by(cluster) %>% top_n(n = 10, wt = avg_log2FC);
pdf(sprintf("%s/heatmap.jpg", outdir), height=20, width=18);
DoHeatmap(scrna_str, features=top10$gene, slot="scale.data", disp.min=-2, disp.max=2,  raster
= FALSE, draw.lines = TRUE, group.by="ident", group.bar=TRUE) +
scale_fill_gradientn(colors = c("blue", "white", "red")) + theme(axis.text.y = element_text(size =
10));


#below plot shows specific gene express in cells
FeaturePlot(scrna, reduction = "tsne", order = TRUE, cols = c("light grey", "red"), pt.size = 0.9,
features = "PGR")
RidgePlot(scrna, features = "HMGB2")
VlnPlot(scrna, features = "HMGB2")
```

```
DotPlot(scrna, features = c("HMGB2", "ANXA8", "TOP2A", "TYMS"), cols = c("light grey", "red")) + RotatedAxis()


DotPlot(scrna, features = c("BIRC5", "CDK6", "CENPF", "CENPW", "FDCSP", "HIST1H4C", "HMGB2", "STMN1", "TOP2A", "TPX2", "TYMS", "UBE2C", "UBE2S", "KRT5", "KRT18", "EPCAM", "VIM"), cols = c("light grey", "red")) + RotatedAxis()


saveRDS(scrna, file = sprintf("%s/Vis2021_7N_12-16_filter_nPC100.rds", outdir));
```

```
#To reload processed data into R later

scrna <- readRDS(file="/users/jmcmullen/Downloads/GSE161529/Vis2021_7N_12-16_filter_nPC100.rds")


# Vis2021_7N_12-16_filter_anchor.rds
# Vis2021_7N_12-16_filter_anchor_0.14 res.rds
```

<u>Code Used to examine triple-negative breast cancer scRNAseq data in R</u>

<u>Start Seurat</u>:

```
library(Seurat)
        library(dplyr)
library(ggplot2)
library(cowplot)
library(Matrix)
library(Biobase)
library(RColorBrewer)
library(data.table)
library(ggthemes)
library(devtools)
library(BH)
library(scClustViz)
library(presto)
library(sctransform)
library(glmGamPoi)
library(limma)
library(GO.db)
library(org.Hs.eg.db)
library(iDEA)



samples = c("TN-MH0126", "TN-MH0135", "TN-SH0106", "TN-MH0114-T2", "TN-B1-
MH4031", "TN-B1-MH0131", "TN-B1-Tum0554", "TN-B1-MH0177")
outdir = "/users/jmcmullen/Downloads/GSE161529";

memory.limit(size=200000)

data.10x = list();
data.10x[[1]] <- Read10X(data.dir
='/users/jmcmullen/Downloads/GSE161529/GSM4909281_TN-MH0126');
data.10x[[2]] <- Read10X(data.dir
='/users/jmcmullen/Downloads/GSE161529/GSM4909282_TN-MH0135');
data.10x[[3]] <- Read10X(data.dir
='/users/jmcmullen/Downloads/GSE161529/GSM4909283_TN-SH0106');
data.10x[[4]] <- Read10X(data.dir
='/users/jmcmullen/Downloads/GSE161529/GSM4909284_TN-MH0114-T2');
data.10x[[5]] <- Read10X(data.dir
='/users/jmcmullen/Downloads/GSE161529/GSM4909285_TN-B1-MH4031');
data.10x[[6]] <- Read10X(data.dir
='/users/jmcmullen/Downloads/GSE161529/GSM4909286_TN-B1-MH0131');
data.10x[[7]] <- Read10X(data.dir
='/users/jmcmullen/Downloads/GSE161529/GSM4909287_TN-B1-Tum0554');
```

```
data.10x[[8]] <- Read10X(data.dir
='/users/jmcmullen/Downloads/GSE161529/GSM4909288_TN-B1-MH0177');

scrna.list = list();
##can remove or change filtering parameters (min.cells=100, min.features=500,)
for (i in 1:length(data.10x)) {scrna.list[[i]] = CreateSeuratObject(counts = data.10x[[i]],
min.cells=100, min.features=500, project=samples[i]); scrna.list[[i]][["DataSet"]] = samples[i];}

rm(data.10x);

scrna <- merge(x=scrna.list[[1]],
y=c(scrna.list[[2]],scrna.list[[3]],scrna.list[[4]],scrna.list[[5]],scrna.list[[6]],scrna.list[[7]],
scrna.list[[8]]), add.cell.ids = c("1","2","3","4","5","6","7","8"), project="VisvaderTN")

rm(scrna.list);

##QC procedures_1



mito.genes <- grep(pattern = "^MT-", x = rownames(x = scrna), value = TRUE);
percent.mito <- Matrix::colSums(x = GetAssayData(object = scrna, slot = 'counts')[mito.genes, ])
/ Matrix::colSums(x = GetAssayData(object = scrna, slot = 'counts'));
scrna[['percent.mito']] <- percent.mito;

ribo.genes <- grep(pattern = "^RP[SL][[:digit:]]", x = rownames(x = scrna), value = TRUE);
percent.ribo <- Matrix::colSums(x = GetAssayData(object = scrna, slot = 'counts')[ribo.genes, ])
/ Matrix::colSums(x = GetAssayData(object = scrna, slot = 'counts'));




#nCount_RNA is the number of unique molecular identifier (UMI) / cell
scrna$log10GenesPerUMI <- log10(scrna$nFeature_RNA) / log10(scrna$nCount_RNA)
#above command creates a ratio between nfeatures/nCount in the metadata

scrna$mitoRatio <- PercentageFeatureSet(object = scrna, pattern = "^MT-")

#extract metadata frame to modify w/o messing up existing metadata
metadata <- scrna@meta.data

metadata <- metadata %>% dplyr::rename(seq_folder = orig.ident, nUMI = nCount_RNA,
nGene = nFeature_RNA)
#change names of features

scrna@meta.data <- metadata
```

```
scrna <- subset(x = scrna, subset= (nUMI >= 1500) & (nGene >= 500) & (log10GenesPerUMI >
0.80) & (percent.mito < 0.20))

##filtering out genes not expressed in any cells- extract counts metric, examine number of cells
##expressing gene and make cutoffs, (10+ cells in this example)
counts <- GetAssayData(object = scrna, slot = "counts")
nonzero <- counts > 0
keep_genes <- Matrix::rowSums(nonzero) >= 10
filtered_counts <- counts[keep_genes, ]
scrna <- CreateSeuratObject(filtered_counts, meta.data = scrna@meta.data)


##END QC
scrna <- PercentageFeatureSet(scrna, pattern = "^MT-", col.name = "percent.mt")
scrna <- SCTransform(scrna, method = "glmGamPoi", vars.to.regress = "percent.mt", verbose =
TRUE)



scrna <- RunPCA(object = scrna, npcs = 100, verbose = FALSE);
scrna <- ProjectDim(object = scrna)

nPC = 100;
scrna <- RunUMAP(object = scrna, reduction = "pca", dims = 1:nPC);
scrna <- RunTSNE(object = scrna, reduction = "pca", dims = 1:nPC);

saveRDS(scrna, file = sprintf("%s/Vis2021_8TN_filter.rds", outdir));



scrna <- FindNeighbors(object = scrna, dims = 1:nPC);
scrna <- FindClusters(object = scrna, resolution = 0.14)

DimPlot(scrna, reduction = "tsne", label = TRUE) + NoLegend()

FeaturePlot(scrna, reduction = "tsne", order = TRUE, cols = c("light grey", "red"), pt.size = 0.9,
features = "PGR")


scrna <- subset(scrna, idents = c("1","2","8","9","10","11"), invert = TRUE)

saveRDS(scrna, file = sprintf("%s/Vis2021_8TN_filter_subset.rds", outdir));



#recluster after subset
```

```
scrna <- RunPCA(object = scrna, npcs = 100, verbose = FALSE);
scrna <- ProjectDim(object = scrna)

nPC = 100;
scrna <- RunUMAP(object = scrna, reduction = "pca", dims = 1:nPC);
scrna <- RunTSNE(object = scrna, reduction = "pca", dims = 1:nPC);

scrna <- FindNeighbors(object = scrna, dims = 1:nPC);
scrna <- FindClusters(object = scrna, resolution = 0.25)

DimPlot(scrna, reduction = "tsne", label = TRUE) + NoLegend()

FeaturePlot(scrna, reduction = "tsne", order = TRUE, cols = c("light grey", "red"), pt.size = 0.9,
features = "PGR")


saveRDS(scrna, file = sprintf("%s/Vis2021_8TN_filter_subset_cluster.rds", outdir));

cluster.breakdown <- table(scrna@meta.data$DataSet, scrna@meta.data$seurat_clusters);

DEGs <- FindAllMarkers(object=scrna, logfc.threshold=1, min.diff.pct=.2);
write.table(DEGs, file=sprintf("%s/DEGs.Wilcox.xls", outdir), quote=FALSE, sep="\t",
row.names=FALSE);

top10 <- DEGs %>% group_by(cluster) %>% top_n(n = 10, wt = avg_log2FC);
pdf(sprintf("%s/heatmap.jpg", outdir), height=20, width=18);
DoHeatmap(scrna_str, features=top10$gene, slot="scale.data", disp.min=-2, disp.max=2,  raster
= FALSE, draw.lines = TRUE, group.by="ident", group.bar=TRUE) +
scale_fill_gradientn(colors = c("blue", "white", "red")) + theme(axis.text.y = element_text(size =
10));

DotPlot(scrna, features = c("BIRC5", "CDK6", "CENPF", "CENPW", "FDCSP", "HIST1H4C",
"HMGB2", "STMN1", "TOP2A", "TPX2", "TYMS", "UBE2C", "UBE2S", "KRT5", "KRT18",
"EPCAM", "VIM"), cols = c("light grey", "red")) + RotatedAxis()



#rename datsets to sample names

scrna@meta.data$DataSet <- plyr::mapvalues(scrna@meta.data$DataSet, c("TN1", "TN2",
"TN3", "TN4", "TN5", "TN6", "TN7", "TN8"), c("TN-MH0126", "TN-MH0135", "TN-
SH0106", "TN-MH0114-T2", "TN-B1-MH4031", "TN-B1-MH0131", "TN-B1-Tum0554", "TN-
B1-MH0177"))

DimPlot(object = scrna, reduction = "tsne", group.by = "DataSet", pt.size=0.1)
```

```
print(plot_grid(p1));
```

```
#To reload processed data into R later

scrna <-
readRDS(file="/users/jmcmullen/Downloads/GSE161529/Vis2021_8TN_filter_subset_cluster.rd
s")

#Vis2021_8TN_filter.rds
# Vis2021_8TN_filter_subset.rds
# Vis2021_8TN_filter_subset_cluster.rds
```
